# Lifestyle Enrichment in Later Life and Its Association With Dementia Risk

**DOI:** 10.1001/jamanetworkopen.2023.23690

**Published:** 2023-07-14

**Authors:** Zimu Wu, Danushika H. Pandigama, Jo Wrigglesworth, Alice Owen, Robyn L. Woods, Trevor T.-J. Chong, Suzanne G. Orchard, Raj C. Shah, Kerry M. Sheets, John J. McNeil, Anne M. Murray, Joanne Ryan

**Affiliations:** 1 School of Public Health and Preventive Medicine, Monash University, Melbourne, Australia; 2 Monash School of Medicine, Monash University, Melbourne, Australia; 3 Turner Institute for Brain and Mental Health, Monash University, Melbourne, Australia; 4 Department of Neurology, Alfred Health, Melbourne, Australia; 5 Department of Clinical Neurosciences, St Vincent’s Hospital, Melbourne, Australia; 6 Department of Family and Preventive Medicine, Rush Alzheimer’s Disease Center, Rush University Medical Center, Chicago, Illinois; 7 Division of Geriatric and Palliative Medicine, Department of Medicine, Hennepin Healthcare, Minneapolis, Minnesota; 8 Berman Center for Outcomes and Clinical Research, Minneapolis, Minnesota

## Abstract

**Importance:**

Lifestyles enriched with socially and mentally stimulating activities in older age may help build cognitive reserve and reduce dementia risk.

**Objective:**

To investigate the association of leisure activities and social networks with dementia risk among older individuals.

**Design, Setting, and Participants:**

This longitudinal prospective cohort study used population-based data from the ASPREE Longitudinal Study of Older Persons (ALSOP) for March 1, 2010, to November 30, 2020. Community-dwelling individuals in Australia aged 70 years or older who were generally healthy and without major cognitive impairment at enrollment were recruited to the ALSOP study between March 1, 2010, and December 31, 2014. Data were analyzed from December 1, 2022, to March 31, 2023.

**Exposures:**

A total of 19 measures of leisure activities and social networks assessed at baseline were classified using exploratory factor analysis.

**Main Outcomes and Measures:**

Dementia was adjudicated by an international expert panel according to *Diagnostic and Statistical Manual of Mental Disorders* (Fourth Edition) criteria. Cox proportional hazards regression examined dementia risk over 10 years, adjusting for education, socioeconomic status, and a range of health-related factors.

**Results:**

This study included 10 318 participants. Their median age was 73.8 (IQR, 71.6-77.2) years at baseline, more than half (52.6%) were women, and most self-identified as White (98.0%). In adusted analyses, more frequent engagement in adult literacy activities (eg, writing letters or journaling, using a computer, and taking education classes) and in active mental activities (eg, playing games, cards, or chess and doing crosswords or puzzles) was associated with an 11.0% (adjusted hazard ratio [AHR], 0.89 [95% CI, 0.85-0.93]) and a 9.0% (AHR, 0.91 [95% CI, 0.87-0.95]) lower risk of dementia, respectively. To a lesser extent, engagement in creative artistic activities (craftwork, woodwork, or metalwork and painting or drawing) (AHR, 0.93 [95% CI, 0.88-0.99]) and in passive mental activities (reading books, newspapers, or magazines; watching television; and listening to music or the radio) (AHR, 0.93 [95% CI, 0.86-0.99]) was also associated with reduced dementia risk. In contrast, interpersonal networks, social activities, and external outings were not associated with dementia risk in this sample.

**Conclusions and Relevance:**

These results suggest that engagement in adult literacy, creative art, and active and passive mental activities may help reduce dementia risk in late life. In addition, these findings may guide policies for geriatric care and interventions targeting dementia prevention for older adults.

## Key Points

**Question:**

Are socially and mentally stimulating activities associated with reduced dementia risk among older adults who have reached age 70 years in relatively good health?

**Findings:**

In this cohort study of 10 318 older individuals in Australia, more frequent participation in adult literacy activities (taking education classes, using a computer, and writing letters or journals) and in active mental activities (playing games, cards, or chess and doing crosswords or puzzles) was associated with reduced dementia risk over 10 years. However, social outings and interactions were not associated with dementia risk.

**Meaning:**

These findings suggest that certain types of cognitively stimulating leisure activities, including adult literacy and active mental activities, may help prevent dementia in older age.

## Teaser

This cohort study examines the association of participation in socially and mentally stimulating activities with dementia risk among generally healthy older adults.

## Introduction

In 2022, there were 55 million individuals worldwide living with dementia, with 10 million new cases emerging annually.^1^ The loss of cognitive function affects the physical and mental health of individuals with dementia and the well-being of their carers and families.^2^ No current treatment approach for dementia has been shown to be completely effective.^1,3^ Therefore, identifying new strategies to prevent or delay dementia onset among older individuals is a priority.

Early-life education is linked to better cognition in later life and reduced dementia risk.^4,5^ Occupation and lifestyle enrichment are also considered cognitive reserve proxies that may help prevent dementia.^4,6^ For older adults, lifestyle enrichment may be particularly important because it could help prevent dementia through modifications to daily routines.^7^ An enriched lifestyle with diverse leisure activities may reflect an optimistic personality^8^ and confer cognitive benefits by stimulating the growth of neurons and synapses^7^ and promoting well-being.^9,10,11,12,13^

Despite accumulating evidence that lifestyle enrichment is associated with better cognitive function and lower dementia risk,^14,15^ only a few studies have investigated participation in social and cognitive activities among older adults. Furthermore, these studies have often focused on individual activities or a single composite score and have not always adjusted for education and health status. Individuals participating in leisure and social activities may be healthier than their peers,^12,13,16,17^ and some activities may offer greater benefits than others. This information is important to support public health recommendations. This study aimed to investigate whether lifestyle enrichment in older relatively healthy individuals is associated with dementia risk, independent of education and health status.

## Methods

### Study Population

This longitudinal prospective cohort study used population-based data from the ASPREE Longitudinal Study of Older Persons (ALSOP) for March 1, 2010, to November 30, 2020. The ALSOP is a substudy exclusively focused on Australian participants recruited for the ASPREE (ASPirin in Reducing Events in the Elderly) study.^18,19^ In brief, community-dwelling individuals aged 70 years or older, without major cognitive impairment (dementia or a Modified Mini-Mental State Examination [3MS] score ≥78) and cardiovascular disease, were recruited through general and family practice providers between March 1, 2010, and December 31, 2014. The ethics review board at each participating institution approved the ASPREE trial. The Monash University Human Research Ethics Committee approved the ALSOP substudy. All participants provided written informed consent. This study followed the Strengthening the Reporting of Observational Studies in Epidemiology (STROBE) reporting guideline.

### Dementia Ascertainment

Individuals with suspected dementia were identified through regular study visits and included (1) those with a low 3MS score (<78) or a substantial decrease in 3MS score (>10 points from a predicted age- and education-adjusted), (2) those with a self-reported or documented (reported in medical or clinical notes) diagnosis of dementia, or (3) those with a prescription for dementia medication. For individuals with suspected dementia, the following measures were administered^20^: the Alzheimer Disease Assessment Scale–Cognitive subscale, the Color Trails Test, the Lurian overlapping figures test, and the Alzheimer Disease Cooperative Study Instrumental Activities of Daily Living Scale; additional documents (eg, laboratory test results, hospital records, specialist reports, or brain imaging) were also collected, when available. Dementia was adjudicated through consensus review of all the above-mentioned documentation, as well as the results of annual cognitive testing (eg, Hopkins Verbal Learning Test–Revised, Controlled Oral Word Association Test, or Symbol Digit Modalities Test), by an international expert panel, according to *Diagnostic and Statistical Manual of Mental Disorders* (Fourth Edition) criteria,^21^ as described previously.^22^

### Lifestyle Enrichment

Information on socially and mentally stimulating activities as well as social networks was obtained through an ALSOP questionnaire, administered during the first year of the ASPREE trial. Questionnaire topics included the following: (1) social networks (number of close relatives and close friends and monthly relative and friend contact [measured using a 10-point Likert scale with 6 categories: 0, 1, 2, 3-4, 5-8, or ≥9]), (2) leisure activities (frequency of participating in club and group activities; taking education classes; using a computer; writing letters or journals; craftwork, woodwork, or metalwork; painting or drawing; playing games, cards, or chess; doing crosswords or puzzles; watching television; listening to music or the radio; and reading books, newspapers, and magazines [measured with a 5-category Likert scale: never, rarely, sometimes, often, or always]), and (3) external outings (frequency of attending libraries; restaurants or cafés; museums, galleries, or exhibitions; and cinemas or theaters [measured with a 5-category Likert scale: never, rarely, sometimes, often, or always]).

### Statistical Analysis

To reduce the number of separate exposure variables being examined (and type I error) and to take into account the correlations between variables, exploratory factor analysis on the 19 leisure activities and social network variables was performed, following prespecified criteria^23^: an eigenvalue of 1 or greater for each factor; a minimum 50% variance explained by the factors extracted; each variable significantly loaded on 1 factor; each factor significantly loaded on by 2 or more variables; and results being theoretically interpretable. An oblique rotation, which allows correlations between factors, was used. Each factor score was computed by summing the values of the variables that had loadings of 0.3 or greater on the corresponding factor.

The association of each factor with the risk of dementia was examined using Cox proportional hazards regression, with Schoenfeld residuals used to assess the proportional hazards assumption. Models adjusted for age, sex, race and ethnicity, education, area-level socioeconomic status (Index of Relative Socio-economic Advantage and Disadvantage), living situation, smoking status, alcohol intake, physical activities, body mass index, hypertension, diabetes, dyslipidemia, depression, and frailty. Full variable definitions are provided in the Table 1 footnotes. These characteristics were compared according to dementia status using the χ^2^ test. Stratified analyses were performed to assess potential sex differences.

**Table 1.** Baseline Characteristics of Included Participants According to Dementia Status Over Follow-Up

Characteristic	No. of participants (%) (N = 10 318)		*P* value^a^
	Without dementia (n = 9972)	With dementia (n = 346)	
Age, y			
70-74	6166 (61.8)	130 (37.6)	<.001
75-84	3514 (35.2)	188 (54.3)	
≥85	292 (2.9)	28 (8.1)	
Sex			
Male	4696 (47.1)	195 (56.4)	.001
Female	5276 (52.9)	151 (43.6)	
Race and ethnicity			
White	9778 (98.0)	336 (97.1)	.22
Other^b^	194 (2.0)	10 (2.9)	
Education, y			
<12	4644 (46.6)	175 (50.6)	.11
12-15	2703 (27.1)	97 (28.0)	
≥16	2625 (26.3)	74 (21.4)	
IRSAD, quintile^c^			
1-4	8045 (80.7)	268 (77.5)	.14
5	1927 (19.3)	78 (22.5)	
Living situation			
Alone at home	7000 (70.2)	235 (67.9)	.36
With someone else	2972 (29.8)	111 (32.1)	
Smoking status			
Current or former	4444 (44.6)	137 (39.6)	.07
Never	5528 (55.4)	209 (60.4)	
Alcohol intake			
Current or former	8537 (85.6)	283 (81.8)	.05
Never	1435 (14.4)	63 (18.2)	
Physical activity			
Rarely or light	3369 (33.8)	129 (37.3)	.40
Moderate	5035 (50.5)	165 (47.7)	
Vigorous	1568 (15.7)	52 (15.0)	
Hypertension^d^			
No	2586 (25.9)	97 (28.0)	.38
Yes	7386 (74.1)	249 (72.0)	
Diabetes mellitus^e^			
No	9036 (90.6)	308 (89.0)	.32
Yes	936 (9.4)	38 (11.0)	
Dyslipidemia^f^			
No	3295 (33.0)	121 (35.0)	.45
Yes	6677 (67.0)	225 (65.0)	
Body mass index^g^			
Underweight or normal	2579 (25.9)	133 (38.4)	<.001
Overweight	4610 (46.2)	137 (39.6)	
Obesity	2783 (27.9)	76 (22.0)	
Depression^h^			
No	9123 (91.5)	303 (87.6)	.01
Yes	849 (8.5)	43 (12.4)	
Frailty^i^			
Nonfrail	6506 (65.2)	172 (49.7)	<.001
Frail or prefrail	3466 (34.8)	174 (50.3)	

^a^ *P* values are based on the Pearson χ^2^ test or Fisher exact test.

^b^ Race and ethnicity was self-reported. Other was defined as any racial and ethnic category with less than 100 participants (including Aboriginal or Torres Strait Islander; Asian; Black; Hispanic or Latino; Native Hawaiian, Pacific Islander, or Māori; or more than 1 race and ethnicity) and those whose race and ethnicity could not be determined.

^c^ The Index of Relative Socio-economic Advantage and Disadvantage (IRSAD) measures socioeconomic conditions according to residential area.

^d^ Hypertension was defined as treatment receipt for high blood pressure or blood pressure greater than 140/90 mm Hg at study entry.

^e^ Diabetes was defined from self-reports or fasting glucose measurements of 126 mg/dL or greater (≥7 mmol/L) or from treatment receipt for diabetes.

^f^ Dyslipidemia was defined as taking cholesterol-lowering medications or having a serum cholesterol level of 212 mg/dL or greater (≥5 mmol/L) or low-density lipoprotein greater than 160 mg/dL (>4.1 mmol/L).

^g^ Body mass index (BMI) was calculated as weight in kilograms divided by height in meters squared and is reported as underweight or normal (<25), overweight (≥25), or obesity (≥30).

^h^ Depression was defined using a Center of Epidemiologic Studies Depression Scale, 10-item version score of 8 or greater.^24^

^i^ Frailty status was defined based on the modified Fried frailty phenotype (including being underweight, weak grip strength, exhaustion, slow walking speed, and low physical activity).^25^

Self-identified race and ethnicity was collected as part of the ASPREE trial as major demographic information. The recruitment criteria for the ASPREEE trial was age 70 years or older but was lowered to 65 years or older for Hispanic and Latino and Black US participants. Thus, it was essential to collect this information to ensure that eligibility criteria were met for the trial. Due to the small sample size of each race and ethnicity in the “other” category (Table 1), participants in these racial and ethnic groups were pooled in the statistical analysis.

Although recruited participants were without major cognitive impairment, to help minimize reverse causality, sensitivity analysis was conducted by (1) excluding individuals diagnosed with dementia, or lost to follow-up, in the first 3 years and (2) adjusting for baseline global cognition (3MS score).

Stata, version 16.0 (StataCorp LLC), was used for analyses, with statistical significance set at *P* < .05 (2-tailed). Data were analyzed from December 1, 2022, to March 31, 2023.

## Results

Our analysis included 10 318 participants with complete information on lifestyle enrichment variables and covariates (eFigure 1 and eTable 1 in Supplement 1 for comparison with excluded participants). The median age was 73.8 (IQR, 71.6-77.2) years; 52.6% of participants were women and 47.4% were men. Most participants (98.0%) self-identified as White; race and ethnicity was self-reported as other or unknown for 2.0%. Table 1 summarizes participant characteristics according to their dementia status over the follow-up period. Individuals with dementia were older, a higher proportion were men, and they were more likely to have lower levels of physical activity and to be in poorer health than individuals without dementia.

Information on the 19 lifestyle enrichment measures is presented in eTable 2 in Supplement 1. Most participants (38.4% and 39.7%, respectively) reported having 3 to 4 relatives and close friends, and they saw their close relatives 5 to 8 times a month (33.4%) and close friends a little less often. Very few participants had no close relatives (2.5%) or close friends (5.2%). In terms of the frequency of leisure activities, watching television, listening to music or the radio, and reading were the most common, with more than 73.5% of participants reporting they always did these activities. More than half of participants (53.9%) reported that they always used computers. In contrast, most participants (75.8%) never drew or painted. For external outings, restaurants and cafés were the most frequently attended (71.4% attended at least sometimes).

The factor analysis revealed a 7-factor solution that accounted for around 70.0% of the total variance in the 19 variables. These factors include interpersonal networks, social activities, adult literacy, creative artistic, active mental, passive mental, and external outings. The variables of each factor and their loadings are presented in Table 2. Number of close friends significantly loaded on 2 factors: interpersonal networks and social activities. However, the loading on the former was much weaker (0.39 vs 0.64), so this variable was only kept for social activities.

**Table 2.** Factor Loadings of Lifestyle Enrichment Factors From the 7-Factor Solution

Lifestyle enrichment factor	Measure (n = 10 517)						
	Interpersonal networks	Social activities	Adult literacy	Creative artistic	Active mental	Passive mental	External outings
Social network^a^							
Close relatives	0.86^b^	0.12	0.02	0.02	0.01	−0.03	0.02
Monthly contact with relatives	0.84^b^	0.03	0.01	0.02	0.02	0.01	0.04
Close friends	0.39	0.64^b^	0.05	0.02	−0.06	0.00	0.01
Monthly contact with friends	0.21	0.75^b^	0.04	0.04	0.01	0.02	0.03
Leisure activity^c^							
Club and group activities	−0.25	0.67^b^	−0.05	0.05	0.17	0.05	0.16
Education classes	−0.17	0.21	0.35^b^	0.27	−0.05	−0.02	0.15
Computer usage	0.03	0.02	0.91^b^	−0.22	0.09	0.03	−0.14
Writing letters or journals	0.05	−0.01	0.78^b^	0.05	−0.03	0.03	0.01
Craft, woodwork, or metalwork	0.09	0.06	−0.20	0.80^b^	0.13	0.05	−0.17
Painting or drawing	−0.03	0.03	0.02	0.73^b^	−0.10	−0.03	0.03
Games, cards, or chess	−0.03	0.19	0.08	−0.02	0.74^b^	−0.14	−0.04
Puzzles or crosswords	0.05	−0.06	0.00	0.04	0.79^b^	0.09	−0.02
Watching television	−0.10	0.15	−0.03	−0.09	0.02	0.68^b^	−0.15
Listening to music or the radio	−0.05	0.06	0.10	0.07	−0.16	0.68^b^	0.04
Reading books, newspapers, or magazines	0.15	−0.19	0.00	0.05	0.18	0.58^b^	0.15
Venue attendance^d^							
Library	−0.03	−0.26	0.17	0.15	0.16	−0.01	0.38^b^
Restaurant or café	0.02	0.09	−0.03	−0.27	0.00	0.02	0.72^b^
Museum, gallery, or exhibition	0.04	−0.05	0.11	0.17	−0.06	−0.06	0.70^b^
Cinema or theater	0.03	0.16	−0.19	−0.10	−0.02	0.03	0.81^b^

^a^ Number of close relatives or close friends and monthly relative or friend contact was measured using a 10-point Likert scale with 6 categories (0, 1, 2, 3-4, 5-8, or ≥9).

^b^ Factor loadings of 0.3 or greater.

^c^ Frequency of engaging in leisure activities was measured using a 5-category Likert scale (never, rarely, sometimes, often, or always).

^d^ Frequency of venue attendance was measured using a 5-category Likert scale (never, rarely, sometimes, often, or always).

The Figure shows the fully adjusted associations between the factor scores and the risk of incident dementia. Increasing the frequency of participation in adult literacy (education classes, computer usage, and writing letters and journals) by 1 category (eg, from sometimes to often) was associated with an 11.0% reduction in dementia risk (adjusted hazard ratio [AHR], 0.89 [95% CI, 0.85-0.93]; *P* < .001). More frequent participation in active mental activities (playing games, cards, or chess or doing puzzles and crosswords) was associated with a 9.0% reduction in the risk of dementia (AHR, 0.91 [95% CI, 0.87-0.96]; *P* < .001). In addition, engagement in more frequent creative artistic activities (AHR, 0.93 [95% CI, 0.88-0.99]; *P* = .03) and in passive mental activities (AHR, 0.93 [95% CI, 0.86-0.99]; *P* = .03) was associated with lower dementia risk.

**Figure.**
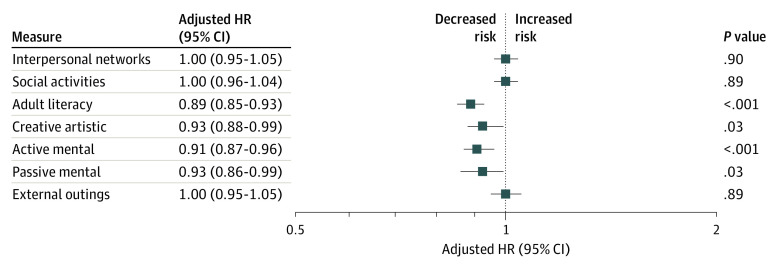
Adjusted Associations Between Lifestyle Enrichment and Incident Dementia for the 10 318 Participants Models adjusted for age (continuous), sex (male or female), race and ethnicity (White or other), education (<12, 12-15, or ≥16 years), socioeconomic status (Index of Relative Socio-economic Advantage and Disadvantage quintile), living situation (at home alone or living with someone else), smoking status (never or former, or current), alcohol intake (never or former, or current), physical activities (rarely or light, moderate, or vigorous), body mass index (underweight or normal, overweight, or obese), hypertension (yes or no), diabetes (yes or no), dyslipidemia (yes or no), depression (yes or no), and Fried frailty phenotype (frailty or prefrailty, or nonfrailty) at baseline. Dementia was diagnosed according to *Diagnostic and Statistical Manual of Mental Disorders* (Fourth Edition) criteria. HR indicates hazard ratio.

In sex-specific analysis (eFigure 2 in Supplement 1), the associations for adult literacy activities (men: AHR, 0.89 [95% CI, 0.84-0.94]; *P* < .001; women: AHR, 0.89 [95% CI, 0.83-0.94]; *P* < .001) and active mental activities (men: AHR, 0.91 [95% CI, 0.85-0.97]; *P* = .005; women: AHR, 0.91 [95% CI, 0.85-0.98]; *P* = .009) with dementia risk remained across both sexes. However, the associations with passive mental activities were no longer present, and the associations with creative artistic activities were only observed for men (AHR, 0.92 [95% CI, 0.85-1.00]; *P* = .05).

For the factors that were associated with dementia risk, we then examined associations of the individual variables that loaded on these factors (eTable 3 in Supplement 1). The frequency of computer usage, writing letters and journals, and playing crosswords and puzzles showed an association with reduced dementia risk across men and women. Participation in craftwork, woodwork, and metalwork was associated with a lower risk of dementia only in men (AHR, 0.88 [95% CI, 0.79-0.97]; *P* = .01). No association was shown for the other individual variables.

The sensitivity analysis did not show major changes from the main analysis (eTables 4 and 5 in Supplement 1). However, the results of passive mental activities were no longer significant after excluding those who developed dementia in the first 3 years or adjusting for baseline cognitive function. Although the findings were also attenuated for creative artistic and active mental activities, the association of adult literacy with dementia remained in all sensitivity analyses.

## Discussion

We observed that certain cognitively stimulating activities were associated with a lower risk of incident dementia among 10 318 older individuals who had reached age 70 years in generally good health and without major cognitive impairment. In particular, a higher frequency of engagement in adult literacy and active mental activities was associated with a 9.0% to 11.0% reduction in dementia risk. With a smaller effect size, creative artistic and passive mental activities both conferred a 7.0% decrease. These associations remained even with the adjustment of key confounders, such as earlier education and socioeconomic status. In contrast, the magnitude of interpersonal networks and the frequency of social activities was not associated with dementia risk. These findings were mostly consistent across sexes and in the sensitivity analyses, which excluded dementia events in the first 3 years. This comprehensive investigation into the association between a range of social and cognitively stimulating activities and dementia risk provides information about modifiable approaches that could help delay the onset of dementia in later life. The inclusion of 10 318 participants with complete data in this cohort enhances the internal validity with a large sample size and minimizes random errors. These strengths addressed the methodological limitations in previous studies but also expanded the knowledge of potential cognitive benefits that can be derived by modification of daily behaviors.

In this study, adult literacy and active mental activities showed the largest associations with reduced risk of dementia, possibly reflecting greater cognitive stimulation. These activities involve proactive engagement, critical thinking, logical reasoning, and social interaction. The cognitive stimulation from such activities can increase resilience against brain pathologies by increasing the number of neurons, enhancing synaptic activity, and permitting higher efficiency in using brain networks.^7^ Adult literacy comprises class attendance, computer usage, and writing—all of which require the processing and storage of new information, which decelerates neurobiological aging^26^ and protects against dementia.^4^ Older individuals who are highly engaged in adult learning may be more likely to use computers and compose written works as a means to fulfill the learning tasks. Writing is a complex process of information output transferring thoughts into texts and using most cognitive abilities.^27^ Although short-term outcomes associated with writing activities on cognitive function remain inconclusive,^28^ our findings suggest potential benefits for dementia prevention over a longer time frame. For computer usage, the operation of the interactive interface requires the coordination of multiple brain regions with motor skills of the fingers (eg, typing and mouse movements). Also, adopting new technologies is a cognitively challenging process that requires learning and practice. This is supported by neuroimaging evidence from a group of individuals aged 55 to 76 years showing the activation of multiple brain regions during internet searching, and the signals were even stronger in those with extensive experience in using the computer and internet.^29^ In addition, 2 studies observed higher cognitive function among older individuals with greater usage of computers.^30,31^

In this study, another factor that was associated with lower dementia risk was active mental activities. This factor includes doing crosswords and puzzles and playing games, cards, or chess, which are generally activities done with others and thus also involve a social interaction component. Many of these activities are competitive in nature and involve complex strategies and problem-solving. They use a variety of cognitive domains, including episodic memory, visuospatial skills, calculation, executive function, attention, and concentration.^32,33^ Crossword puzzles may also utilize language skills and semantic memory that help solve verbal and linguistic problems using preexisting knowledge. Our findings are consistent with previous studies demonstrating that performing these activities is associated with less decline in general cognitive function and several cognitive domains (eg, memory, verbal fluency) among older individuals without dementia.^34,35^ Studies investigating these activities in relation to dementia have also reported associations,^9,36^ although not consistently. For example, one study using data from more than 8000 individuals aged an average of 56 years failed to find an association between playing games, cards, bingo, and chess and 18-year dementia risk.^10^ However, crosswords and puzzles were not included in their questionnaire, and participants were almost 20 years younger at baseline than the ASPREE and ALSOP participants.

Our findings may also provide information for cognitive interventions. There have been many cognitive training programs targeting older individuals. The ACTIVE (Advanced Cognitive Training for Independent and Vital Elderly) study, which is one of the earliest large-scale trials in this field, examined the efficacy of supervised training in memory, reasoning, and processing speed respectively in 3 groups, and domain-specific improvements were observed immediately and 5 years after the initial intervention.^37^ More recently, several smaller-scale trials have also been implemented. One trial observed greater cognitive improvements in those receiving multidomain training compared with the control participants who performed occupational tasks (eg, drawing, reading, gardening, etc).^38^ This is in line with our findings that proactive manipulation of knowledge may have a greater contribution to dementia prevention than recreational activities. A few trials also found that cognitive training, delivered by either computers or robots, was beneficial to cognitive function,^39,40,41^ which echoes the present results on computer usage. However, the true benefits of lifestyle interventions may be smaller than anticipated. Prior studies, for example, have demonstrated smaller overall benefits of exercise training on dementia risk^42^ compared with the benefits anticipated from epidemiological evidence.^43^ Individuals who routinely participate in leisure activities may possess certain behavioral and personality attributes, which are not sufficiently adjusted or controlled for in the analysis. Therefore, despite neurostructural changes after cognitive training,^44^ the extent to which certain leisure activities could be translated into true benefits in dementia prevention remains inconclusive and further research is needed.

In general, we did not observe sex differences in these associations. The only exception was creative artistic activities (handicrafts, particularly), which were only marginally associated with reduced dementia risk in men. One possible reason for this is that the types of handiwork preferred by men in this study may be more physically demanding (eg, woodworking or metalworking). These activities generally involve the use of heavy tools and materials, thus being more laborious and possibly conferring greater benefits from physical activity. Also, these activities themselves may be cognitively stimulating, since the psychosocial benefits of crafting for mental well-being have been shown previously.^45,46^ Therefore, it may be promising to advocate gender-neutral handiwork in communities for older individuals.

Unexpectedly, social activities and interpersonal networks were not associated with dementia risk. Social cohesion has been considered a protective factor against dementia^47,48^ and a well-established protective factor against cognitive impairment.^49,50^ Our findings may be driven by the fact that most participants were well engaged socially, with only a small proportion being lonely or isolated.^51,52^ As such, our analysis largely compared individuals with moderate and large social networks. It is plausible that the current participants who all remained cognitively intact into later life have already built cognitive reserve to some extent via prior life experiences. Thus, it may be reasonable to tailor the strategies of cognitive aging according to social engagement and health status to maximize the use of health resources.

### Limitations

This study had some limitations. First, selection bias may have affected these findings. Individuals consenting to involvement in this study were likely more amenable to scientific research, and the inclusion criteria were such that participants were healthier at enrollment compared with the general population. Also, those with complete data were possibly more confident in reading and completing forms. As such, compared with the wider community, the included participants may have had healthier lifestyles in general and were more likely to be engaged with their community already. This, combined with the lack of racially and ethnically diverse participants, limits the generalizability of the findings. Second, despite the various socially and cognitively stimulating activities examined here, other factors that may benefit cognitive health, such as watching sports events or speaking non–first languages, were not available in the questionnaire.^53,54^ Third, we cannot rule out reverse causality, although we undertook additional analysis to help control for it. Participants recruited to the ASPREE study were without major cognitive impairment at baseline, and we ran additional sensitivity analysis excluding participants diagnosed with dementia in the first 3 years of the study. However, dementia has a long prodromal period, and it remains possible that behavioral changes that precede dementia symptoms even by several years may help explain some of the findings shown here.

## Conclusions

In this cohort study with 10 318 older community-dwelling individuals, participation in adult literacy activities and in active mental activities was associated with reduced risk of incident dementia. These associations remained after taking into account education and socioeconomic status, which are known to be associated with cognitive function and lifestyle, and largely remained in sensitivity analyses. These findings can help inform strategies for dementia prevention and cognitive reserve strengthening in later life, in the context of modifiable daily routines.

## Notes

Supplement 1. **eTable 1.** Characteristics of the Excluded and Included Participants (n = 16 703) **eTable 2.** Number of Participants by Number of Relatives and Friends Close or Contacted Monthly, and Frequency of Leisure Activities and Venue Attendance (N = 10 318) **eTable 3.** Fully Adjusted Associations Between Individual Variables That Were Significant in the Factor Analysis and Incident Dementia (N = 10 318) **eTable 4.** Adjusted Associations Between Lifestyle Enrichment and Incident Dementia, Excluding Dementia Cases and Participants Otherwise Censored in the First 3 Years After Baseline (n = 10 006) **eTable 5.** Adjusted Associations Between Lifestyle Enrichment and Incident Dementia (N = 10 318) **eFigure 1.** Flow Chart of Study Participants **eFigure 2.** Adjusted Associations Between Lifestyle Enrichment and Incident Dementia by Men (n = 4889) and Women (n = 5429)

Supplement 2.
Data Sharing Statement
