# Supplementary material for: Lifestyle Enrichment in Later Life and Its Association With Dementia Risk
Source: JAMA Netw Open. 2023 Jul 14;6(7):e2323690. doi: 10.1001/jamanetworkopen.2023.23690 (PMC10349343; doi:10.1001/jamanetworkopen.2023.23690)
Supplement: Supplement 1. — eTable 1. Characteristics of the Excluded and Included Participants (n = 16 703) eTable 2. Number of Participants by Number of Relatives and Friends Close or Contacted Monthly, and Frequency of Leisure Activities and Venue Attendance (N = 10 318) eTable 3. Fully Adjusted Associations Between Individual Variables That Were Significant in the Factor Analysis and Incident Dementia (N = 10 318) eTable 4. Adjusted Associations Between Lifestyle Enrichment and Incident Dementia, Excluding Dementia Cases and Participants Otherwise Censored in the First 3 Years After Baseline (n = 10 006) eTable 5. Adjusted Associations Between Lifestyle Enrichment and Incident Dementia (N = 10 318) eFigure 1. Flow Chart of Study Participants eFigure 2. Adjusted Associations Between Lifestyle Enrichment and Incident Dementia by Men (n = 4889) and Women (n = 5429) [file jamanetwopen-e2323690-s001.pdf]

Supplemental Online Content

Wu Z, Pandigama DH, Wrigglesworth J, et al. Lifestyle enrichment in later life and its association with dementia risk. *JAMA Netw Open*. 2023;6(7):e2323690. doi:10.1001/jamanetworkopen.2023.23690

eTable 1. Characteristics of the Excluded and Included Participants (n = 16 703)

eTable 2. Number of Participants by Number of Relatives and Friends Close or Contacted Monthly, and Frequency of Leisure Activities and Venue Attendance (N = 10 318)

eTable 3. Fully Adjusted Associations Between Individual Variables That Were Significant in the Factor Analysis and Incident Dementia (N = 10 318)

eTable 4. Adjusted Associations Between Lifestyle Enrichment and Incident Dementia, Excluding Dementia Cases and Participants Otherwise Censored in the First 3 Years After Baseline (n = 10 006)

eTable 5. Adjusted Associations Between Lifestyle Enrichment and Incident Dementia (N = 10 318)

eFigure 1. Flow Chart of Study Participants

eFigure 2. Adjusted Associations Between Lifestyle Enrichment and Incident Dementia by Men (n = 4889) and Women (n = 5429)

This supplemental material has been provided by the authors to give readers additional information about their work.

© 2023 Wu Z et al. *JAMA Network Open*.

eTable 1. Characteristics of the Excluded and Included Participants (n = 16 703)

Baseline characteristics (No. %)	Excluded * (n=6,385)	Included (n=10,318)	P-value [a]
**Age, years**			
70-74	3,373 (52.8)	6,296 (61.0)	<0.001
75-84	2,692 (42.2)	3,702 (35.9)	
≥85	320 (5.0)	320 (3.1)	
**Gender**			
Men	2,633 (41.2)	4,891 (47.4)	<0.001
Women	3,752 (58.8)	5,427 (52.6)	
**Ethnicity**			
White	6,248 (97.9)	10,114 (98.0)	0.45
Other [b]	137 (2.1)	204 (2.0)	
**Education, years**			
<12	3,580 (56.1)	4,819 (46.7)	<0.001
12-15	1,579 (24.7)	2,800 (27.1)	
≥16	1,225 (19.2)	2,699 (26.2)	
**IRSAD [c]**			
1st to 4th quintile	5,214 (82.3)	8,313 (80.6)	<0.01
5th quintile	1,123 (17.7)	2,005 (19.4)	
**Living situation**			
Alone at home	2,248 (35.2)	3,083 (29.9)	<0.001
With someone	4,137 (64.8)	7,235 (70.1)	
**Smoking status**			
Current/Former	2,832 (44.3)	4,581 (44.4)	1.0
Never	3,553 (55.7)	5,737 (55.6)	
**Alcohol intake**			
Current/Former	5,177 (81.1)	8,820 (85.5)	<0.001
Never	1,208 (18.9)	1,498 (14.5)	
**Physical activity**			
Rarely/Light	800 (33.8)	3,498 (33.9)	0.99
Moderate	1,197 (50.5)	5,200 (50.4)	
Vigorous	373 (15.7)	1,620 (15.7)	
**Hypertension [d]**			
No	1,495 (23.4)	2,683 (26.0)	<0.001
Yes	4,890 (76.6)	7,635 (74.0)	
**Diabetes mellitus [e]**			
No	5,715 (89.5)	9,344 (90.6)	0.03
Yes	670 (10.5)	974 (9.44)	
**Dyslipidemia [f]**			
No	1,977 (31.0)	3,416 (33.1)	<0.01
Yes	4,408 (69.0)	6,902 (66.9)	
**Body mass index [g]**			
Underweight/Normal	1,665 (26.4)	2,712 (26.3)	<0.01
Overweight	2,736 (43.4)	4,747 (46.0)	
Obese	1,906 (30.2)	2,859 (27.1)	
**Depression [h]**			
No	5,678 (89.0)	9,426 (91.4)	<0.001
Yes	703 (11.0)	892 (8.7)	
**Frailty [i]**			
Non-frail	3,614 (56.6)	6,678 (64.7)	<0.001
Frail/Pre-frail	2,771 (43.4)	3,640 (35.3)	

* The numbers do not necessarily add up to 6,385 due to incomplete data.

[a] P-values are based on Pearson's chi-squared test or Fisher's exact test.

[b] Other ethnicities were defined as any ethnical category with less than 100 participants, including Black, Hispanic/Latino, Aboriginal/Torres Strait Islanders, Native Hawaiian/Pacific Islander/Maori, Asian, more than one race, and those whose ethnicity could not be determined.

[c] IRSAD refers to the Index of Relative Socio-economic Advantage and Disadvantage, which measures the socioeconomic conditions according to residential areas.

[d] Hypertension was defined as on treatment for high blood pressure or blood pressure >140/90 mmHg at study entry.

[e] Diabetes was defined from self-report or fasting glucose ≥126mg/dL (≥7 mmol/L) or on treatment for diabetes.

© 2023 Wu Z et al. *JAMA Network Open*.

[f] Dyslipidemia was defined as those taking cholesterol-lowering medications or serum cholesterol ≥212 mg/dL (≥5 mmol/L; Australia) or LDL > 160 mg/dL (>4.1 mmol/L).
[g] Underweight was defined as body mass index ≤ 18.5 kg/m^2, overweight as body mass index ≥25 kg/m^2 and obesity as body mass index ≥30 kg/m^2.
[h] Depression was defined as Center of Epidemiologic Studies Depression Scale, 10-item version (CES-D-10) ≥8.
[i] Frailty status was defined based on the modified Fried frailty phenotype (including being underweight, weak grip strength, exhaustion, slow walking speed, and low physical activity).

© 2023 Wu Z et al. *JAMA Network Open*.

eTable 2. Number of Participants by Number of Relatives and Friends Close or Contacted Monthly, and Frequency of Leisure Activities and Venue Attendance (N = 10 318)

	No. (%)					
**Social networks, number**	**0**	**1**	**2**	**3-4**	**5-8**	**≥9**
Close relatives	253 (2.5)	534 (5.2)	1,452 (14.1)	3,964 (38.4)	2,737 (26.5)	1,378 (13.4)
Relatives in contact with monthly	148 (1.4)	351 (3.4)	797 (7.7)	2,949 (28.6)	3,450 (33.4)	2,623 (25.4)
Close friends	537 (5.2)	652 (6.3)	1,972 (19.1)	4,095 (39.7)	2,011 (19.5)	1,051 (10.2)
Friends in contact with monthly	182 (1.8)	206 (2.0)	697 (6.8)	2,554 (24.8)	3,046 (29.5)	3,633 (35.2)
**Leisure activities, frequency**	**Never**	**Rarely**	**Sometimes**	**Often**	**Always**	
Club and group activities	1,679 (16.3)	1,310 (12.7)	2,290 (22.2)	4,587 (44.5)	452 (4.4)	
Education classes	6,861 (66.5)	2,065 (20.0)	624 (6.1)	741 (7.2)	27 (0.3)	
Computer usage	2,582 (25.0)	438 (4.3)	435 (4.2)	1,299 (12.6)	5,564 (53.9)	
Writing letters/journals	1,783 (17.3)	2,904 (28.1)	2,257 (21.9)	2,168 (21.0)	1,206 (11.7)	
Craft/woodwork/metalwork	2,454 (23.8)	1,961 (19.0)	1,662 (16.1)	2,110 (20.5)	2,131 (20.7)	
Painting/drawing	7,824 (75.8)	1,587 (15.4)	412 (4.0)	343 (3.3)	152 (1.5)	
Games/cards/chess	4,013 (38.9)	3,110 (30.1)	1,169 (11.3)	1,303 (12.6)	723 (7.0)	
Puzzles/crosswords	2,533 (24.6)	1,497 (14.5)	901 (8.7)	1,445 (14.0)	3,942 (38.2)	
Watching television	33 (0.3)	47 (0.5)	126 (1.2)	969 (9.4)	9,143 (88.6)	
Listening to music/radio	168 (1.6)	488 (4.7)	551 (5.3)	1,531 (14.8)	7,580 (73.5)	
Reading books/newspapers/magazines	29 (0.3)	120 (1.2)	248 (2.4)	1,128 (10.9)	8,793 (85.2)	
**Venue attendance, frequency**	**Never**	**Rarely**	**Sometimes**	**Often**	**Always**	
Library	3,577 (34.7)	2,950 (28.6)	2,487 (24.1)	1,184 (11.5)	120 (1.2)	
Restaurant/café	288 (2.8)	2,668 (25.9)	4,269 (41.4)	2,851 (27.6)	242 (2.4)	
Museum/gallery/exhibition	2,079 (20.2)	6,026 (58.4)	1,959 (19.0)	231 (2.2)	23 (0.2)	
Cinema/theatre	1,131 (11.0)	4,996 (48.4)	3,228 (31.3)	924 (9.0)	39 (0.4)	

© 2023 Wu Z et al. *JAMA Network Open*.

eTable 3. Fully Adjusted[a] Associations Between Individual Variables That Were Significant in the Factor Analysis and Incident Dementia[b] (N = 10 318)

Lifestyle enrichment	Overall		Men (n=4,891)		Women (n=5,427)	
	HR (95% CI)	P-value	HR (95% CI)	P-value	HR (95% CI)	P-value
Education classes	0.94 (0.82-1.07)	0.35	1.04 (0.87-1.25)	0.64	0.84 (0.69-1.02)	0.08
Computer usage	0.83 (0.78-0.89)	<0.001	0.82 (0.75-0.89)	<0.001	0.84 (0.76-0.93)	<0.01
Writing letters and journals	0.83 (0.75-0.91)	<0.001	0.81 (0.72-0.93)	<0.01	0.84 (0.73-0.97)	0.02
Craftwork/woodwork/metalwork	0.91 (0.84-0.98)	0.01	0.88 (0.79-0.97)	0.01	0.93 (0.84-1.04)	0.23
Painting/drawing	0.97 (0.85-1.12)	0.70	1.03 (0.86-1.24)	0.74	0.92 (0.75-1.13)	0.43
Games/cards/chess	0.93 (0.85-1.02)	0.11	0.91 (0.80-1.04)	0.17	0.94 (0.83-1.07)	0.36
Crosswords and puzzles	0.87 (0.82-0.93)	<0.001	0.88 (0.81-0.96)	<0.01	0.86 (0.78-0.95)	<0.01
Watching television	0.91 (0.75-1.10)	0.32	0.93 (0.72-1.20)	0.58	0.87 (0.64-1.18)	0.37
Listening to music/radio	0.91 (0.82-1.00)	0.06	0.92 (0.80-1.05)	0.23	0.88 (0.75-1.03)	0.10
Reading books/newspapers/magazines	0.91 (0.76-1.07)	0.25	0.85 (0.70-1.03)	0.10	1.05 (0.74-1.49)	0.77

Abbreviations: HR, hazard ratio; CI, confidence interval

Note: one unit of increase in each factor refers to one level up in the frequency of the corresponding activities.

[a] The models adjusted for age (continuous), gender (men; women), ethnicity (white; other), education (<12 years; 12-15 years; ≥16 years), socioeconomic status (Index of Relative Socio-economic Advantage and Disadvantage: quintiles), living situation (at home alone; at home with someone or in a residential home), smoking status (never/former; current), alcohol intake (never/former; current), physical activities (rarely/light; moderate; vigorous), body mass index (underweight/normal; overweight; obese), hypertension (yes; no), diabetes (yes; no), dyslipidemia (yes; no), depression (yes; no), and Fried frailty phenotype (frailty/pre-frailty; non-frailty) at baseline.

[b] Dementia was diagnosed according to the criteria of the Diagnostic and Statistical Manual of Mental Disorders, fourth edition.

© 2023 Wu Z et al. *JAMA Network Open*.

eTable 4. Adjusted[a] Associations Between Lifestyle Enrichment and Incident Dementia, Excluding Dementia Cases and Participants Otherwise Censored in the First 3 Years After Baseline[b] (n = 10 006)

Lifestyle enrichment	Overall		Men (n=4,711)		Women (n=5,295)	
	HR (95% CI)	P-value	HR (95% CI)	P-value	HR (95% CI)	P-value
**Minimally adjusted**						
Interpersonal networks [c]	1.00 (0.94-1.06)	0.90	0.98 (0.91-1.06)	0.69	1.01 (0.92-1.10)	0.88
Social activities [d]	1.01 (0.96-1.05)	0.81	0.99 (0.94-1.05)	0.75	1.03 (0.96-1.10)	0.48
Adult literacy [e]	0.91 (0.87-0.95)	<0.001	0.93 (0.87-0.99)	0.02	0.88 (0.82-0.94)	<0.001
Creative artistic [f]	0.92 (0.86-0.99)	0.02	0.92 (0.83-1.00)	0.06	0.93 (0.84-1.03)	0.15
Active mental [g]	0.91 (0.86-0.96)	<0.01	0.91 (0.84-0.98)	0.01	0.92 (0.85-0.99)	0.03
Passive mental [h]	0.94 (0.87-1.02)	0.16	0.94 (0.85-1.04)	0.25	0.95 (0.83-1.09)	0.45
External outings [i]	1.03 (0.97-1.09)	0.36	1.02 (0.94-1.10)	0.66	1.04 (0.96-1.12)	0.37
**Maximally adjusted**						
Interpersonal networks [c]	1.00 (0.95-1.07)	0.90	0.99 (0.91-1.07)	0.72	1.01 (0.93-1.11)	0.78
Social activities [d]	1.01 (0.97-1.06)	0.59	0.99 (0.94-1.05)	0.78	1.04 (0.97-1.12)	0.29
Adult literacy [e]	0.91 (0.87-0.96)	<0.001	0.93 (0.87-0.99)	0.02	0.90 (0.84-0.96)	<0.01
Creative artistic [f]	0.94 (0.87-1.00)	0.06	0.92 (0.84-1.01)	0.10	0.94 (0.85-1.04)	0.22
Active mental [g]	0.93 (0.88-0.98)	<0.01	0.92 (0.85-0.99)	0.02	0.93 (0.86-1.01)	0.09
Passive mental [h]	0.95 (0.88-1.03)	0.23	0.94 (0.85-1.04)	0.25	0.97 (0.84-1.11)	0.66
External outings [i]	1.03 (0.98-1.09)	0.24	1.01 (0.94-1.10)	0.71	1.06 (0.98-1.15)	0.17

Abbreviations: HR, hazard ratio; CI, confidence interval

Note: 1) one unit of increase in each factor refers to one level up in the frequency of external outings or leisure activities, or one more friend or relative close or contract monthly, 2) the analysis excluded the dementia cases that occurred within the first three years after enrolment.

[a] The minimally adjusted models adjusted for age (continuous), gender (men; women), ethnicity (white; other), education (<12 years; 12-15 years; ≥16 years), socioeconomic status (Index of Relative Socio-economic Advantage and Disadvantage: quintiles); The fully adjusted model included all variables in the minimally adjusted model and further adjusted for living situation (at home alone; at home with someone or in a residential home), smoking status (never/former; current), alcohol intake (never/former; current), physical activities (rarely/light; moderate; vigorous), body mass index (underweight/normal; overweight; obese), hypertension (yes; no), diabetes (yes; no), dyslipidemia (yes; no), depression (yes; no), and Fried frailty phenotype (frailty/pre-frailty; non-frailty) at baseline.

[b] Dementia was diagnosed according to the criteria of the Diagnostic and Statistical Manual of Mental Disorders, fourth edition.

[c] Interpersonal networks involves the number of friends and relatives close, and relatives contact monthly.

[d] Social activities involves the number of friends close and contact monthly, and the frequency of attending group activities and clubs.

[e] Adult literacy involves the frequency of attending education classes, using a computer and writing journals and letters.

[f] Creative artistic involves the frequencies of doing craftwork/woodwork/metalwork and painting/drawing.

[g] Active mental involves the frequencies of playing games/cards/chess and doing puzzles and crosswords.

[h] Passive mental involves the frequencies of watching television, listening to music/radio and reading books/newspapers/magazines.

[i] External outings involves the frequency of visiting library, restaurant/café, museums/galleries/exhibitions, cinema and theatre.

© 2023 Wu Z et al. *JAMA Network Open*.

eTable 5. Adjusted[a] Associations Between Lifestyle Enrichment and Incident Dementia[b] (N = 10 318)

Lifestyle enrichment	Overall		Men (n=4,891)		Women (n=5,427)	
	HR (95% CI)	P-value	HR (95% CI)	P-value	HR (95% CI)	P-value
**Minimally adjusted**						
Interpersonal networks [c]	1.00 (0.95-1.05)	0.95	0.98 (0.92-1.05)	0.62	1.03 (0.95-1.11)	0.47
Social activities [d]	1.00 (0.96-1.04)	0.98	0.98 (0.94-1.03)	0.53	1.03 (0.97-1.10)	0.36
Adult literacy [e]	0.92 (0.88-0.96)	<0.001	0.92 (0.87-0.97)	<0.01	0.92 (0.87-0.98)	<0.01
Creative artistic [f]	0.93 (0.88-0.99)	0.02	0.92 (0.85-1.00)	0.04	0.95 (0.87-1.03)	0.22
Active mental [g]	0.95 (0.90-0.99)	0.04	0.94 (0.88-1.01)	0.09	0.96 (0.89-1.03)	0.24
Passive mental [h]	0.94 (0.88-1.01)	0.11	0.95 (0.87-1.04)	0.24	0.94 (0.84-1.05)	0.27
External outings [i]	1.02 (0.97-1.07)	0.51	1.01 (0.94-1.08)	0.79	1.02 (0.96-1.10)	0.51
**Fully adjusted**						
Interpersonal networks [c]	1.01 (0.95-1.06)	0.82	0.98 (0.92-1.06)	0.67	1.04 (0.98-1.10)	0.23
Social activities [d]	1.01 (0.97-1.05)	0.77	0.99 (0.94-1.04)	0.57	1.04 (0.97-1.11)	0.25
Adult literacy [e]	0.93 (0.90-0.97)	<0.001	0.92 (0.87-0.98)	<0.01	0.94 (0.88-0.99)	0.03
Creative artistic [f]	0.95 (0.89-1.00)	0.07	0.93 (0.86-1.01)	0.09	0.96 (0.87-1.04)	0.31
Active mental [g]	0.96 (0.91-1.01)	0.10	0.95 (0.89-1.02)	0.15	0.97 (0.90-1.04)	0.39
Passive mental [h]	0.95 (0.89-1.02)	0.19	0.96 (0.88-1.04)	0.31	0.95 (0.85-1.07)	0.42
External outings [i]	1.02 (0.97-1.07)	0.48	1.01 (0.94-1.08)	0.89	1.03 (0.95-1.12)	0.43

Abbreviations: HR, hazard ratio; CI, confidence interval

Note: 1) one unit of increase in each factor refers to one level up in the frequency of external outings or leisure activities, or one more friend or relative close or contract monthly, 2) the analysis excluded the dementia cases that occurred within the first three years after enrolment.

[a] The minimally adjusted models adjusted for age (continuous), gender (men; women), ethnicity (white; other), education (<12 years; 12-15 years; ≥16 years), socioeconomic status (Index of Relative Socio-economic Advantage and Disadvantage: quintiles); The fully adjusted model included all variables in the minimally adjusted model and further adjusted for living situation (at home alone; at home with someone or in a residential home), smoking status (never/former; current), alcohol intake (never/former; current), physical activities (rarely/light; moderate; vigorous), body mass index (underweight/normal; overweight; obese), hypertension (yes; no), diabetes (yes; no), dyslipidemia (yes; no), depression (yes; no), Fried frailty phenotype (frailty/pre-frailty; non-frailty) and global cognitive function (Modified Mini-Mental State Examination, quintiles) at baseline.

[b] Dementia was diagnosed according to the criteria of the Diagnostic and Statistical Manual of Mental Disorders, fourth edition.

[c] Interpersonal networks involves the number of friends and relatives close, and relatives contact monthly.

[d] Social activities involves the number of friends close and contact monthly, and the frequency of attending group activities and clubs.

[e] Adult literacy involves the frequency of attending education classes, using a computer and writing journals and letters.

[f] Creative artistic involves the frequencies of doing craftwork/woodwork/metalwork and painting/drawing.

[g] Active mental involves the frequencies of playing games/cards/chess and doing puzzles and crosswords.

[h] Passive mental involves the frequencies of watching television, listening to music/radio and reading books/newspapers/magazines.

[i] External outings involves the frequency of visiting library, restaurant/café, museums/galleries/exhibitions, cinema and theatre.

© 2023 Wu Z et al. *JAMA Network Open*.

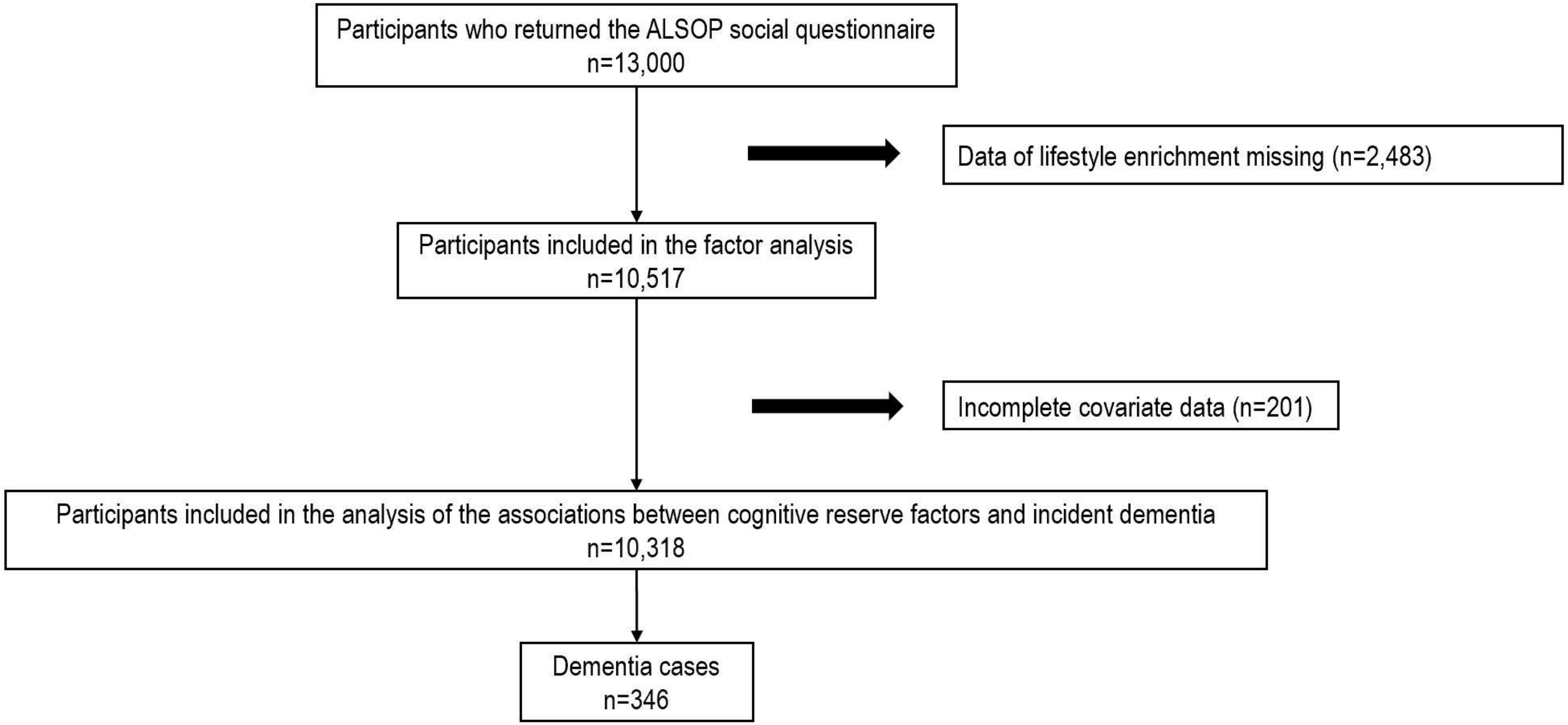

eFigure 1. Flow Chart of Study Participants

© 2023 Wu Z et al. *JAMA Network Open*.

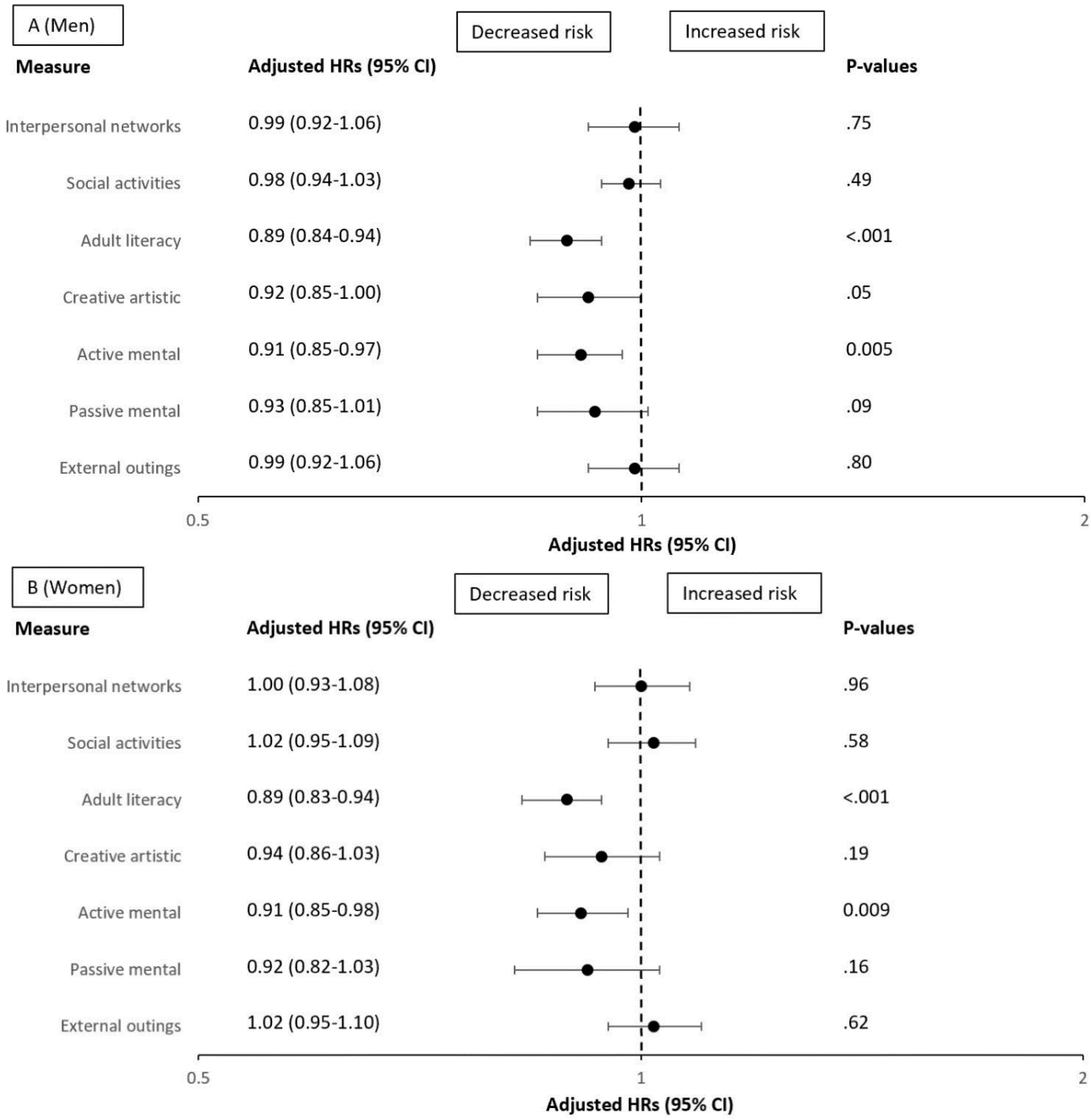

eFigure 2. Adjusted Associations Between Lifestyle Enrichment and Incident Dementia by Men (n = 4889 [A]) and Women (n = 5429 [B])

Abbreviations: HR, hazard ratio; CI, confidence interval; Note: 1) The models adjusted for age (continuous), gender (men; women), ethnicity (white; other), education (<12 years; 12-15 years; ≥16 years), socioeconomic status (Index of Relative Socio-economic Advantage and Disadvantage: quintiles), living situation (at home alone; at home with someone or in a residential home), smoking status (never/former; current), alcohol intake (never/former; current), physical activities (rarely/light; moderate; vigorous), body mass index (underweight/normal; overweight; obese), hypertension (yes; no), diabetes (yes; no), dyslipidemia (yes; no), depression (yes; no), and Fried frailty phenotype (frailty/pre-frailty; non-frailty) at baseline. 2) Dementia was diagnosed according to the criteria of the Diagnostic and Statistical Manual of Mental Disorders, fourth edition.

© 2023 Wu Z et al. *JAMA Network Open*.
