# Supplementary material for: Lifestyle Enrichment in Later Life and Its Association With Dementia Risk
Source: JAMA Netw Open. 2023 Jul 14;6(7):e2323690. doi: 10.1001/jamanetworkopen.2023.23690 (PMC10349343; doi:10.1001/jamanetworkopen.2023.23690)
Supplement: Supplement 2. — Data Sharing Statement [file jamanetwopen-e2323690-s002.pdf]

Data Sharing Statement

Wu. Lifestyle Enrichment in Later Life and Its Association With Dementia Risk. *JAMA Netw Open*. Published July 14, 2023. doi:10.1001/jamanetworkopen.2023.23690

Data
Data available: Yes
How to access the data: Data from the ASPREE study are available upon reasonable request. Please refer to the study website https://ams.aspree.org.
